# Life-Threatening Clostridial Sepsis in a Postmenopausal Patient with Degenerating Uterine Leiomyoma

**DOI:** 10.1155/2010/541959

**Published:** 2010-05-30

**Authors:** Christopher S. Bryant, Latoya Perry, Jay P. Shah, Sanjeev Kumar, Gunter Deppe

**Affiliations:** Division of Gynecologic Oncology, Department of Obstetrics and Gynecology, Barbara Ann Karmanos Cancer Institute, Wayne State University, Detroit, MI 48201, USA

## Abstract

*Clostridium perfringens* is a fulminant infection that affects patients with a high rate of morbidity and mortality. Fortunately, *C. perfringens*-associated sepsis and death in the gynecologic patient is rarely encountered. We report a case of intrauterine *C. perfringens* presenting as life-threatening sepsis in a postmenopausal patient.

## 1. Introduction

Before abortion was legalized in the United States in 1973, reports of *Clostridium perfringens* (formally known as *Clostridium welchii*) commonly occurred in gynecologic patients and resulted in devastating consequences [1]. Although the incidence has markedly decreased, mortality rates continue to remain high with deaths reported in 10% to 85% of patients presenting with *C. perfringens *sepsis [1–3]. 

## 2. Case Report

A 60-year-old female presented to emergency services (ER) reporting a 6-hour history of myalgia, suprapubic pain, and febrile morbidity (38.9°C/102°F). The patient was considered to be of her normal state of health prior to the onset of symptoms. Her gynecologic history was noncontributory and her medical comorbidities consisted of stable hypertension and chronic obstructive pulmonary disease. Initial significant physical examination findings and laboratory results were as follows: lower abdominal pain without rebound or guarding and leukocytosis (WBC 22,000/mm^3^ and 39% bands). The patient was initially admitted with sepsis (not otherwise specified) to the general medicine service and was prescribed intravenous antimicrobial therapy (ampicillin sodium/sulbactam sodium). Within 6 hours after admission, the patient complained of increasing abdominopelvic pain and developed clinical signs of sepstic shock. Intravenous gentamycin and clindamycin were added to the antimicrobial regimen, and the patient was transferred to the medical intensive care unit (MICU). Computed tomography (CT) revealed normal abdominal anatomy except for a uterus measuring 21 cm × 16 cm with fluid within the endometrial cavity and multiple leiomyomas (no evidence of myometrial gas). Gynecology was consulted and further examination confirmed a pelvic mass with suprapubic and cervical motion tenderness. After bedside endometrial biopsy was unable to be performed secondary to cervical stenosis, an ultrasound-guided transvaginal endometrial biopsy was performed as previously described [4]. The patient's condition continued to worsen and clinical management was complicated by the development of anemia, hyperbilirubinemia, hemoglobinemia, hemoglobinuria, and hypotension requiring blood transfusion and vasoactive support. Gram stain of the intrauterine fluid revealed gram-positive rods. Because of evidence suggestive of an intrauterine clostridial infection, an emergent total abdominal hysterectomy and bilateral salpingooophorectomy was performed without operative complications. Intraoperative findings revealed an enlarged fibroid uterus with inflammatory changes involving the uterine serosa, no evidence of tubo-ovarian abscess or infection involving the extrauterine structures. Postoperatively, the patient recovered fully after 4 days of MICU support and 13 days of hospitalization. Final pathology of the surgical specimen revealed a degenerating leiomyoma with intramural myonecrosis without evidence of malignancy. Final culture of blood and intrauterine specimens revealed *Clostridium perfringens*.

## 3. Discussion

After conducting a MEDLINE inquiry of English-language citations for the years 1970 through 2009, using the search phrases “clostridium and postmenopausal,” “clostridium and uterine,” “clostridium and leiomyoma,” and “clostridium and gynecologic cancer,” we identified 5 cases (Table 1) of middle-aged women (Age > 45 years) treated for uterine *Clostridium perfringens-* (previously known as *C. welchii*) associated sepsis. This is the second case, in 35 years, of *C. perfringens* sepsis complicating a degenerating uterine leiomyoma.

*Clostridium perfringens* is an anaerobic, gram-positive rod-shaped organism, that, through the production of extracellular toxins (mainly alpha), produces characteristic local and systemic effects resulting in myonecrosis, shock, and potential death [8]. *C. perfringens* has been isolated from the vagina and cervix of 1% to 10% of healthy women and is considered to be of no clinical significance in the absence of infection [2]. Only about 5% of *C. perfringens *isolates actually cause a clinical illness [9]. 

Clostridial infections complicating obstetrics and gynecology patients are uncommon, but have been associated with obstetrical and gynecologic conditions and/or treatments [2, 5–7, 9–11]. *C. perfringens* infection appears to be rare in middle-aged women (age > 45 years). Our report is the 5th case described in the literature, and there have been no reports of *C. perfringens* infection associated with middle-aged women without instrumentation of the uterus or coexisting uterine pathology.

As seen in our patient, the clinical presentation of *C. perfringens* is commonly associated with multisystem involvement (hemolysis, hemoglobinuria, jaundice, uterine tenderness, and septic shock accompanied by renal failure) [2]. Although not demonstrated in our patient, radiologic studies demonstrating gas patterns involving the myometrium are highly suspicious for uterine *C. perfringens *infection. However, intrauterine gas pattern can be associated with other conditions that may be treated with a more conservative approach [2, 10]. 

Reductions in mortality appear to rely on promptness in diagnosis, supportive care, broad spectrum antibiotics, and surgical debridement. Timely, adequate antimicrobial therapy is important for successful treatment of *C. perfringens* infections. Zaharet al.[3] reported a decrease in the mortality from 63% to 14% with prompt adequate antimicrobial therapy. Penicillin is considered the drug of choice; however, in vitro susceptibility data has demonstrated that combination antimicrobial therapy with penicillin and clindamycin or tetracycline may further reduce mortality through the reduction in toxin synthesis [12, 13]. Hysterectomy without surgical resection of the adnexal structures may be inadequate because of rapid *C. perfringens *involvement of extrauterine structures [1]. If prompt recognition and surgical treatment identify tubes and ovaries without evidence of necrosis, the conservation of the adnexal structures may not confer a poorer outcome [14]. Conservative treatment with supportive care and broad spectrum antibiotics (no surgical treatment) is less commonly performed. A case series reported by Lichtenbergand Henning[9] described no mortality among women with clostridial infection and conservative treatment. However, the authors appropriately explained that none of the patients in their series manifested any signs of sepsis. Hyperbaric oxygen (HBO) has been used to treat *C. perfringens* infections with varying efficacy and the role of HBO remains controversial [2, 15]. There is one report of refractory *C. perfringens* infection that responded to HBO after intravenous antibiotics and hysterectomy failed to control the infection [11]. HBO may offer an improvement in outcome when used in combination with antimicrobial therapy and surgery. However, HBO has not been studied in a prospective manner and may be associated with significant complications [2].

 Due to the limited number of reports of middle-aged women treated for *C. perfringens* infection, it is difficult to identify clinical characteristics that may categorize patients into surgical or nonsurgical treatment groups. Also because of the proclivity for rapid deterioration with *C. perfringens* infection, it may be clinically difficult to allow for a significant length of treatment time to assess the efficacy of the antimicrobial therapy. In our review of the literature (Table 1), 60% of middle-aged women with *C. perfringens *infection died of their condition. Although clinical signs of multisystem involvement were reported in all of the cases, hemolysis was reported in 66% of patients with death as an outcome. Only our patient demonstrated hemolysis and survived treatment. This may be explained by the early presentation of the patient, the use of broad-spectrum antibiotics, and the early timing of antimicrobial and surgical treatment [2]. Although some advocate dilatation and curretage for patients without myometrial gas pattern on imaging studies, the presence of hemolysis, as seen in our patient, may be associated as a potential indicator for poorer outcome and need for more aggressive treatment [2].

In conclusion, *C. perfringens* is a rare condition affecting middle-aged women. This infection appears to be more commonly associated with women with a history of a uterine procedure or underlying pathology. Prompt recognition and treatment are essential for an improved survival.

## Figures and Tables

**Table 1:** Summary of cases of *Clostridium perfringens* in middle-aged (age > 45 years) women.

Reference	Age	Uterine pathology	Clinical conditions^a^	X-Ray^b^	Surgery	Death
Kaufmannet al.[5]	56	Leiomyoma	Fever	Yes^c^	No^d^	Yes
			Hemolysis/anemia			
			Hyperbilirubinemia			
			Hemoglobinemia			
			Hemoglobinuria			
			Hypotension			

Symondsand Robertson[6]	57	Endometrial adenocarcinoma	Fever	No	No	Yes
			Hemolysis/anemia			
			Hyperbilirubinemia			
			Hemoglobinemia			
			Hemoglobinuria			

	49	Endometrial adenocarcinoma	Fever	No	No	No
			Hyperbilirubinemia			

Bravermanet al.[7]	64	Endometrial adenocarcinoma	Fever	Yes	Yes	No
			Anemia^c^			
			Hyperbilirubinemia			

Bryant	60	Leiomyoma	Fever	Yes	Yes	No
			Hemolysis/anemia			
			Hyperbilirubinemia			
			Hemoglobinemia			
			Hemoglobinuria			
			Hypotension			

^a^Clinical conditions are a summary of the laboratory and physical exam findings reported in the manuscript

^b^X-ray includes plain film, sonogram, or computed tomography

^c^X-ray was performed postmortem

^d^Uterus was examined during autopsy.
